# Deep Sequencing of Subseafloor Eukaryotic rRNA Reveals Active Fungi across Marine Subsurface Provinces

**DOI:** 10.1371/journal.pone.0056335

**Published:** 2013-02-13

**Authors:** William Orsi, Jennifer F. Biddle, Virginia Edgcomb

**Affiliations:** 1 Department of Geology and Geophysics, Woods Hole Oceanographic Institution, Woods Hole, Massachusetts, United States of America; 2 College of Earth, Ocean, and Environment, University of Delaware, Lewes, Delaware, United States of America; Université Paris Sud, France

## Abstract

The deep marine subsurface is a vast habitat for microbial life where cells may live on geologic timescales. Because DNA in sediments may be preserved on long timescales, ribosomal RNA (rRNA) is suggested to be a proxy for the active fraction of a microbial community in the subsurface. During an investigation of eukaryotic 18S rRNA by amplicon pyrosequencing, unique profiles of Fungi were found across a range of marine subsurface provinces including ridge flanks, continental margins, and abyssal plains. Subseafloor fungal populations exhibit statistically significant correlations with total organic carbon (TOC), nitrate, sulfide, and dissolved inorganic carbon (DIC). These correlations are supported by terminal restriction length polymorphism (TRFLP) analyses of fungal rRNA. Geochemical correlations with fungal pyrosequencing and TRFLP data from this geographically broad sample set suggests environmental selection of active Fungi in the marine subsurface. Within the same dataset, ancient rRNA signatures were recovered from plants and diatoms in marine sediments ranging from 0.03 to 2.7 million years old, suggesting that rRNA from some eukaryotic taxa may be much more stable than previously considered in the marine subsurface.

## Introduction

The deep marine subsurface harbors an immense number of microbes, initially observed by microscopy and later confirmed by nucleic acid and lipid studies [1]–[5]. The Bacteria and Archaea of the subsurface appear to be mostly heterotrophic [3], [6], surviving on organic matter originally derived from photosynthesis, performing metabolic functions such as sulfate reduction, methanogenesis, and fermentation [7]. Biomass turnover likely proceeds on the order of thousands of years in the marine subsurface [8], affecting large-scale biogeochemical cycling over geological timescales. Recent studies of subsurface microbial metabolisms e.g. [3], [9], [10] and communities e.g. [1], [2], [11]–[14] have provided a foundation for understanding the role of subsurface microbes. Despite recent advances, there is still little knowledge about which microbes are responsible for most of the activity [3], as past studies have been plagued by methods that either showed conflicting results of diversity [2], [15] or utilized biomolecules with debatable preservation potential [1], [16].

Historically, most sequence-based studies of microbial diversity within environmental samples, including the marine subsurface, have utilized PCR amplification of target genes, most commonly small subunit ribosomal RNA (SSU rRNA), from DNA extracted from the environmental sample as a starting material e.g. [9], [17]. Because these extracts can include DNA from active cells, inactive but viable cells, dead cells, and extracellular DNA from lysed or degraded cells, which can be bound to mineral grains [18], DNA pools do not exclusively represent living organisms. Due to the relative stability and higher preservation potential of DNA, reverse transcription of rRNA followed by PCR amplification is now a common proxy for living/metabolically active microbes in SSU rRNA based environmental surveys. This approach was recently used to target active microbes in marine subsurface samples [2], [19]–[21].

An rRNA-based approach is especially warranted for subsurface molecular investigations given documentation of DNA paleomes in subsurface samples. DNA paleomes are thought to consist of both extracellular DNA and DNA within inactive cells that have been preserved in the marine subsurface. Extracellular DNA and DNA preserved in structures such as cysts, spores, and pollen in sediments are thought to represent one of the largest reservoirs of DNA in the world [22], [23]. This DNA originates from deposition of dissolved DNA, DNA bound to detritus, cysts, endospores, intact pollen, cellular exudates and excretion following cell death, protistan grazing, and viral lysis [23]. Permanently anoxic sediments that contain a high organic matter load are known to promote DNA accumulation in the subsurface [23]. Corinaldesi *et al*. (2011) found increased copy numbers of eukaryotic ribosomal rRNA genes in deeper sediments of the Black Sea, suggesting periods of enhanced primary productivity followed by deposition on the seafloor and progressive accumulation in the sediments. Such DNA paleomes are a sedimentary record of past microbial communities [24], and have been reported and employed to understand paleoenvironments and succession of species as a result of environmental change in core samples of various ages. These include 100 million year old continental drilled black shale [24], [25], terrestrial [26], deep marine sediment [27], [28], and lacustrine environments [29].

A preliminary eukaryotic rRNA-based study of the deep biosphere found fungal rRNA signatures dominated samples from the Peru Margin and Peru Trench. However, a few sequences in the cDNA pool were from unexpected eukaryotes such as animals, green algae, and red algae [21]. Other evidence for Fungi in these habitats [11], [30], suggests that Fungi, with their recognized ability to utilize a wide range of organic substrates for growth [31], could contribute to important large-scale elemental cycles in the marine subsurface. However, in previous studies, signals from subsurface inhabitants unlikely to be active (*i.e.* algae) were not fully investigated. As such, we undertook a survey of a globally distributed subsurface sample set using an rRNA-based amplicon pyrosequencing approach to detect the 18S rRNA of eukaryotes in a much broader subsurface sample set representing several major oceanic provinces. Using stringent contamination controls, we examined the taxonomic distribution of eukaryotes, particularly Fungi, and their relationships to geochemical factors, allowing us to discern potentially active, versus potentially preserved, organisms in a subsurface pool of environmental rRNA.

## Materials and Methods

### Sample collection

Subsurface sediment samples from Hydrate Ridge (IODP Leg 204 Site 1244a; 44° 35′ 17″ N, 125° 07′ 19″ W), Peru Margin (IODP Leg 201 Site 1227a; 79° 57′ 349″ W, 08° 59′ 463″ S), and Eastern Equatorial Pacific (IODP Leg 201 Site 1225a; 110° 43′ 289″ W, 02° 46′ 247″ N) were obtained from the Gulf Coast Core Repository (University of Texas A&M) (Table 1). Gravity core subsurface samples from North Pond (Table 1) near the Mid-Atlantic Ridge (22° 48′ 04″ E, 46° 06′ 30″ N) and Benguela Upwelling System (14° 15′ 04″ E, 27° 44′ 40″ S) were collected on March 3^rd^, 2009 onboard the R/V Maria Merian and April 21^st^, 2008 onboard the R/V Meteor, respectively and were provided by Andreas Teske (University of North Carolina, Chapel Hill, NC). Careful precautions were taken during sampling to avoid contamination during the sampling process. For IODP cores, contamination tests were performed using Perfluorocarbon tracers and fluorescent microspheres (for more information see http://www-odp.tamu.edu/publications/201_IR and http://www-odp.tamu.edu/publications/204_IR). Sediment samples were immediately frozen at −80°C after sampling and stored at −80°C until RNA was extracted. Sediment samples at a sediment depth of 0.01 and 0.08 mbsf from Little Sippewissett Salt Marsh were taken November 13^th^ 2011 using a sterile syringe. Sulfide was detectable in both samples and thus samples were presumed anoxic. No specific permits were required for the described field studies. The locations sampled are not privately owned or protected and field studies did not involve endangered or protected species.

**Table 1:** Samples examined in this study with corresponding metadata.

	Subseafloor samples					Near surface samples	
Site	North Pond [44], [45]	Hydrate Ridge [52]	Benguela Upwelling System [43], [53]–[55]	Eastern Equatorial Pacific [56]	Peru Margin [57]	Sippewissett [58]–[60]	
Sample code	NP	HR	BSP	EEP	PM	SIP1	SIP8
Depth (mbsf)	1.6	1.8	4.6	45.3	48.1	0.01	0.08
Sampling site	GeoB 13507-1	IODP 1244a	GeoB 12805-1	IODP 1225a	IODP 1227a	Great Sippewissett Salt Marsh	
O_2_ (µM)	120	0	0	0	0	150	0
Sulfide (µM)	0	1000	3000	0	6130	175	300
TOC (% wt)	0.2	1.5	3.8	0.01	3.6	24	24
DIC (mM)	2.15	40	12	3	24	4	4
NO_3_^−^ (µM)	34	0	0	0	0	1.5	1.5

### RNA extraction

RNA was extracted from 25 grams of sediment using the FastRNA Pro Soil-Direct Kit (MP Biomedicals, Solon, OH) in a laminar flow hood to reduce contamination from aerosols. Extractions were performed at Woods Hole Oceanographic Institution. Several modifications were made to the protocol provided with the kit to increase RNA yield from low biomass subseafloor samples. It was necessary to scale up the volume of sediment that is typically extracted with the kit (∼0.5 grams) due to the expected low biomass of subsurface eukaryotes. Four 15 ml Lysing Matrix E tubes (MP Biomedicals, Solon, OH) were filled with 5 g sediment and 5 ml of Soil Lysis Solution (MP Biomedicals, Solon, OH). Tubes were vortexed to suspend the sediment and Soil Lysis Solution was added to the tube leaving 1 ml of headspace. Tubes were then homogenized for 60 seconds at a setting of 4.5 on the FastPrep-24 homogenizer (MP Biomedicals, Solon, OH). Contents of the 15 ml tubes were combined into two RNAse free 50 ml falcon tubes and centrifuged for 30 minutes at 4,000 RPM. The supernatants were combined in a new 50 ml RNAse-free falcon tube and 1/10 volume of 2M Sodium Acetate (pH 4.0) was added. An equal volume of phenol-chloroform (pH 6.5) was added and vortexed for 30 seconds, incubated for 5 minutes at room temperature, and centrifuged at 4000 RPM for 20 minutes at 4°C. The top phase was carefully transferred to a new 50 ml falcon tube and 2.5x volumes 100% ethanol and 1/10 volume 3M Sodium Acetate were added and incubated overnight at −80°C. After incubation, tubes were centrifuged at 4000 RPM for 60 minutes at 4°C and the supernatant removed. Pellets were washed with 70% ethanol, centrifuged for 15 minutes at 4°C, and air-dried. Dried pellets were resuspended with 0.25 ml RNAse-free sterile water and combined into a new 1.5 ml RNAse-free tube. 1/10 volume of 2M Sodium Acetate (pH 4.0) and an equal volume of phenol:chloroform (pH 6.5) were added, the tube was vortexed for 1 minute, and incubated for 5 minutes at room temperature. The tube was then centrifuged for 10 minutes at 4°C, the top phase removed into a new RNAse free 1.5 ml tube, and 0.7 volumes of 100% isopropanol was added and incubated for 1hour at −20°C. After incubation tubes were centrifuged for 20 minutes at 14,000 RPM at 4°C and the supernatant was removed. Pellets were washed with 70% ethanol and centrifuged at 14,000 RPM for 5 minutes at 4°C. Ethanol was removed and the pellets air-dried. Pellets were resuspended with 200 µl of RNAse free sterile water and DNA was removed using the Turbo DNA-free kit (Life Technologies, Grand Island, NY). DNAse incubation times were increased to 1hour to ensure removal of contaminating DNA. Samples were then taken through the protocol supplied with the FastRNA Pro Soil-Direct kit to the end (starting at the RNA Matrix and RNA Slurry addition step), including the optional column purification step to remove residual humic acids. To further purify the RNA, we used the MEGA-Clear RNA Purification Kit (Life Technologies, Grand Island, NY). Extraction blanks were performed (adding sterile water instead of sample) to identify aerosolized contaminants that may have entered sample and reagent tubes during the extraction process. To reduce contamination, all RNA extractions were performed in a laminar flow hood.

### RT-PCR amplification of eukaryotic rRNA

To amplify the V4 hypervariable region of eukaryotic rRNA, we used PCR primers targeting this region: EukV4F (5′ – CGTATCGCCTCCCTCGCGCCATCAGxxxxxxxxxx**CCAGCASCYGCGGTAATTCC** – 3′) and EukV4R (5′ – CTATGCGCCTTGCCAGCCCGCTCAG **ACTTTCGTTCTTGATYRA** – 3′), where the x region represents the unique MID barcode used for each sample, the linker primer sequence is underlined, and the 18S rRNA eukaryotic primer is bold. These primers were chosen because they target a wide range of eukaryotic taxa [32]. RT-PCR was performed using the SuperScript One-Step RT-PCR with Platinum Taq kit (Life Technologies, Grand Island, NY). Individual reactions consisted of 2 μl RNA template, 25 μl buffer, 1 μl of forward Primer, 1 μl of reverse primer, 2 μl of the Platinum RT-Taq enzyme mix, and 18 μl RNAse free sterile water. The cDNA step was performed at 55°C and cDNA was amplified in 40 cycles of PCR with an annealing temperature of 65°C (55°C for 30 minutes, 95°C for 5 minutes, [95°C for 15 seconds, 65°C for 30 seconds, 68°C for 1 minute]x40, 68°C for 5 minutes). To check for DNA carryover during the RNA extraction protocol, a separate PCR reaction (at the same number of cycles) was included in which Taq polymerase was substituted for the reverse-transcriptase/platinum Taq enzyme mix. For each sample, 5–10 RT-PCR reactions were performed and extracted using the Zymo Research Gel Extraction Kit (Zymo Research, Irvine, CA). A gel volume of 100% isopropanol was added to each dissolved gel slice before addition to the DNA collection column. Dissolved gel slices from each sample were pooled by centrifuging them all through the same DNA collection column. cDNA was quantified fluorometrically prior to 454 sequencing using the Qubit 2.0 (Life Technologies, Grand Island, NY). To identify contaminants we performed additional RT-PCR amplifications at 55 cycles using RNAse free sterile water and RNA extraction blanks (resulting from RNA extractions in which no sample was added) as template. Contaminants were amplified with primers containing a unique MID in 55 cycles of PCR.

### Quality control, clustering, and taxonomic assignment of 454 data

cDNA amplicons were sequenced on a GS-FLX Titanium 454 sequencer at EnGenCore (University of South Carolina, Columbia, SC), which resulted in ∼37,000 reads. To reduce homopolymer errors inherent to 454 sequencing, the dataset was put through the denoise protocol as described in the QIIME software package [33] using the denoise_wrapper.py command. After denoising, chimeric sequences were identified and removed using ChimeraSlayer with the blast_fragments method in QIIME. The data were subjected to quality score filtering using the split_libraries.py command and clustered at various levels of sequence identity (80%, 85%, 90%, 93%, 95%, 97%) in QIIME using the uclust method of all-to-all pair-wise comparisons via the pick_otus.py command.

The QIIME taxonomy classification pipeline was not able to accurately classify the majority of eukaryotic OTUs. Thus, we used Jaguc, a program developed specifically for classification of eukaryotic rRNA sequence data, to classify our sequence reads [34]. 90% of eukaryotic OTUs were classified to genus using this approach. OTU tables were created using the make_otu_table.py command in QIIME and the Jaguc taxonomy for each OTU was amended onto this table using a custom perl script developed by the authors for this purpose. This perl script is available from the authors upon request.

### Terminal Restriction Fragment Length Polymorphism (TRFLP) analysis of fungal rRNA

To further investigate the fungal diversity in our samples, we used a TRFLP approach using PCR primers specific to fungal 18S rRNA. The fungal primers used were EF3 (5′ – TCCTCTAAATGACCAAGTTTG – 3′) and Fung5 (5′ – GTAAAAGTCCTGGTTCCCC – 3′) [35]. The forward primer, EF3, was labeled with the phosphoramidite dye 6-Carboxyfluorescein (6-FAM) at the 5′-end (Integrated DNA Technologies, Coralville, Iowa). Fungal rRNA was amplified using a cDNA incubation step at 50°C followed by 40 cycles of PCR with an annealing temperature of 53°C. Three RT-PCR reactions were performed for each sample, gel extracted, and pooled using the same protocol as above. Fungal rRNA amplicons were digested with three different restriction enzymes: MspI, RsaI, and HhaI (New England Biolabs, Ipswich, MA), for 1 hr at 37°C. These restriction enzymes were chosen because they have been shown to provide statistically significant TRFLP data for interpreting fungal community structure across different samples [36]. Digests were mixed with the Applied Biosystems size marker GS600LIZ and HiDi Formamide in the ratio 1:1:9 and run on an Applied Biosystems 3730 DNA analyzer (Applied Biosystems, Carlsbad, CA). Electropherograms were analyzed using the PeakScanner software package (Applied Biosystems, Carlsbad, CA) to identify the size, height, and peak area of each T-RF. T-REX [37] was used to filter out noise from true peaks and to align peaks.

### Statistical Analyses

**Figure 1:**
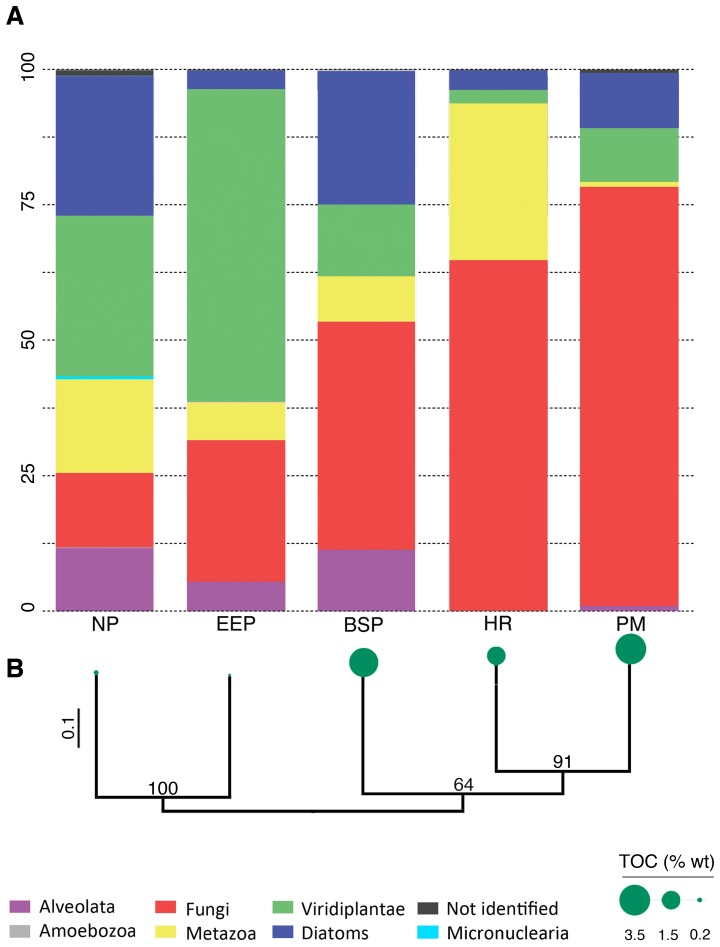
Alpha and beta diversity of pyrosequenced eukaryotic rRNA amplicons from five subseafloor sites representing various marine subsurface provinces. (a) Kingdom and phylum-level affiliation of eukaryotic rRNA sequences. Percentage of total reads is presented on the y-axis, samples were normalized to the number of sequences detected in the sample with the smallest number of sequences prior to comparison and UniFrac. (b) Hierarchical clustering of the eukaryotic rRNA dataset and phylogenetic distances between samples was calculated using weighted UniFrac. Bootstrap support values are shown on nodes. Green circles at the end of the branches are proportional to TOC values. See Table 1 for sample information.

Canonical Correspondence Analysis (CCA) was used to elucidate relationships between eukaryotic community structure and concentrations of dissolved oxygen (O_2_), nitrate (NO_3_^−^) dissolved inorganic carbon (DIC), total organic carbon (TOC), and sulfide. Multi-response Permutation Procedure (MRPP) was used to test for a statistically significant influence of sediment depth, DIC, sulfide, TOC, and oxygen on the observed OTU distributions. All ordination and multivariate statistical analyses were performed on the TRFLP and pyrosequenced datasets as a whole, as well as the five major eukaryotic subgroups that dominated our 454 dataset: Metazoa, Viridiplantae, Diatoms, Alveolates, and Fungi. Analyses were performed on sequences affiliated with these groups clustered at 80, 85, 90, 93, and 97% sequence identity thresholds as well as the fungal TRFLP dataset. MRPP and CCA were implemented using the PC-ORD software package (MjM Software Design). Weighted UniFrac analysis was performed in QIIME [33]. Prior to UniFrac and alpha-diversity comparisons (*i.e.* Fig. 1), the number of sequences per sample were normalized to the sample with the least number of sequences by randomly selecting a subset of sequences from each sample using the multiple_rarefactions.py script in QIIME.

## Results and Discussion

Amplicon libraries of eukaryotic V4 rRNA were prepared from sediments from the Eastern Equatorial Pacific (45.3 mbsf), Peru Margin (48.1 mbsf), Hydrate Ridge (1.8 mbsf), North Pond (1.6 mbsf), the Benguela Continental Slope (4.6 mbsf), and Sippewissett Salt Marsh (0.01 and 0.08 mbsf) (Table 1). Over 20,000 eukaryotic rRNA pyrotags passed quality control (54% of total raw reads), corresponding to 671 operational taxonomic units (OTUs) sharing 97% sequence identity. The majority of sequences that passed quality control affiliated with sequences that are potential contaminants (Table S1, S2) and hence, to present the most conservative picture of subsurface communities, these were removed from downstream analyses (Table S1, S2). Analysis of the remaining data revealed the presence of Diatoms (653 sequences), Viridiplantae (2095 sequences), Metazoa (512 sequences), Alveolata (275 sequences), and Fungi (2559 sequences) within the subseafloor samples (Table 2, Fig. S1). The most abundant taxonomic groups were detected in all samples, with the exception of alveolates, which were detected in 4 out of 5 samples (Fig. 1A). Despite the use of 454 pyrosequencing, rarefaction analysis indicated that saturation was not reached for some subseafloor samples, particularly North Pond (Fig. S2). However, we acknowledge that conclusions regarding coverage of diversity should be made with caution in the absence of replication for individual samples.

**Table 2:** Eukaryotic genera affiliated with rRNA sequences detected in subsurface samples.

Sample	Diatoms	Viridiplantae	Metazoa	Alveolata	Fungi	Sediment age
North Pond (1.6mbsf)	*Nitzschia, Cymbella, Caloneis, Fistulifera, Navicula, Fallacia, Craticula, Stauroneis, Amphora, Staurosirella, Pierrecomperia, Mayamaea*	*Chlamydomonas, Chloroidium, Nannochloris, Chlorella, Acetabularia, Acrochaete, Verdigellas, Bryum, Rosulabryum, Funaria, Cedrus, Plantago, Fallopia, Polygonum, Pisum, Lithocarpus, Juglans, Camelina, Festuca*	*Eudrilus, Thalassophagacarus, Praunus, Tylobolus, Gyratrix*	*Colpodella, Chromerida, Plagiopyla, Frontonia, Strombidium, Loxodes, Cryptocaryon, Amphidinium, Gyrodinium, Prorocentrum, Protodinium*	*Powellomyces, Peyronellaea, Cordyceps bassiana, Apioplagiostoma, Cyberlindnera, Mycena, Hyphodontia, Antrodia, Stereum, Filobasidium, Cryptococcus, Hannaella, Erythrobasidium, Doassansia,*	∼ 0.1–2mya [45]
Hydrate Ridge (1.8mbsf)	*Amphora, Plagiostriata, Staurosirella*	*Plantago, Camelina, Klebsormidium*	*Neoramia, Zealanapis, Heliophanus, Alopecosa, Ethmolaimus, Synonchus*		*Cordyceps bassiana, Entoloma, Crinipellis, Mycena, Cryptococcus, Leucosporidium,*	∼ 0.1mya [52]
Benguela Upwelling System (4.61 mbsf)	*Cymbella, Fistulifera, Fallacia, Amphora, Plagiostriata, Licmophora*	*Plantago, Festuca, Klebsormidium*	*Onychiurus*	*Cystoisospora, Arcuospathidium*	*Acidomyces, Neurospora, Sordaria, Candida, Hydropus, Mycena, Steccherinum, Stereum, Filobasidium, Rhodotorula, Rhodosporidium, Diversispora, Glomus*	∼ 0.03mya [43]
Eastern Equatorial Pacific (45.3mbsf)	*Nitzschia, Navicula, Stauroneis, Chaetoceros, Fragilaria*	*Scenedesmus, Chlorella, Juniperus, Plantago, Pisum, Juglans, Camelina, Allium, Festuca, Klebsormidium*	*Onychiurus, Dicyrtomina, Ethmolaimus, Synonchus, Syringolaimus*	*Cystoisospora, Colpodella, Sorogena, Arcuospathidium, Trimyema, Pseudovorticella*	*Peyronellaea, Alternaria, Camarops, Apioplagiostoma, Cryptosporella, Discula, Phruensis, Neurospora, Candida, Cyberlindnera, Mycena, Antrodia, Rhizoctonia, Filobasidium, Cryptococcus, Dioszegia, Sterigmatomyces, Helicogloea, Rhodotorula, Sporobolomyces,*	∼ 2.77mya [38]
Peru Margin (48.1mbsf)	*Mayamaea*	*Dilabifilum, Bracteacoccus, Chlorella, Plantago, Pisum, Juglans, Festuca, Klebsormidium*	*Entomobrya, Dicyrtomina, Deroceras*	*Colpodella*	*Knufia, Glyphium, Geopyxis, Lentinula, Mycena, Cryptococcus, Trichosporon, Rhodotorula*	∼ 2.6mya [61]

Photosynthetic microbes, higher plants, and multicellular animals are unlikely to be living inhabitants of the deep subsurface in sediments that have been buried for up to 2 million years (Fig. 1A, Table 2). As such, the detection of plant, diatom, and probably metazoan rRNA suggests that rRNA from some eukaryotes is preserved in deep marine sediments. The detection of rRNA from these organisms cannot be explained solely by contamination, due to our extensive controls.

We minimized seawater contamination by sampling the inside of sediment cores using a sterile spatula, which also avoided the outer regions in contact with sampling equipment. Additionally, the average pore size of these sediments is less than 1 µm, which represents a barrier to potential contaminating eukaryotic cells from seawater during the sampling and drilling process. We minimized aerosol contamination (given the low eukaryotic biomass typical of subsurface samples [15]) introduced during RNA extraction and RT-PCR amplification (see Materials and Methods) by sequencing a control generated from 55 cycles of PCR amplification of the eukaryotic V4 hypervariable region using PCR-grade water processed through an unused RNA extraction kit. Contaminant sequences were removed from our subsurface datasets by clustering all the data together into OTUs sharing 97% sequence identity. All OTUs containing a sequence present in the contaminant sample were removed from downstream analyses. These contaminating sequences were identified as originating from fungal, plant, and metazoan genera (Table S2). This removes potentially ubiquitous contaminating organisms and results in a more conservative picture of the subsurface population.

**Figure 2:**
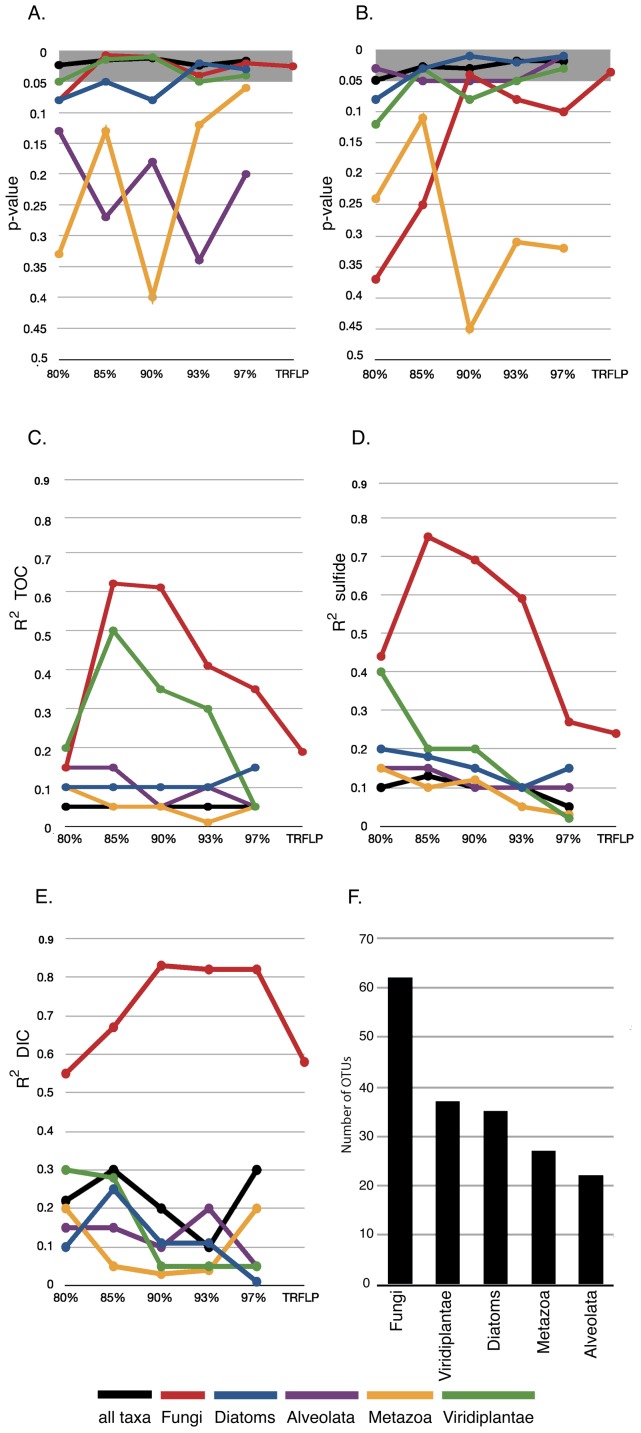
Multi-Response Permutation Procedure (MRPP) and Canonical Correspondence Analysis (CCA) performed on eukaryotic rRNA OTUs sharing 80–97% sequence identity, as well as fungal TRFLP patterns (denoted as ‘TRFLP’ on the right hand side of each plot). (A) MRPP analysis of the combined effect of TOC, DIC, and sulfide and (B) sediment depth on subsurface eukaryotic distributions. Statistically significant p-values (<0.05) are highlighted. CCA correlation values (R^2^) of TOC (C), sulfide (D), and DIC (E) with different eukaryotic taxa in subsurface samples. (F) OTU richness of eukaryotic taxa detected in marine subsurface samples.

Despite attempts at removing contaminants, it is possible that 100% of contaminants were not removed, and that some still remain in our sequence data. We acknowledge this possibility because the Viridiplantae sequences detected are consistent with temperate North American taxa and or cosmopolitan taxa that are common in temperate North America. Also, the Viridiplantae sequences detected do not include many diagnostic taxa for some of the sample locations. However, interpretation of fine-scale taxonomic assignments based on BLASTn is complicated by the hypervariable nature of the V4 region of eukaryotic rRNA, and hence, precise taxonomic assignments are often difficult, certainly at the species, and sometimes genus level. For this reason, we clustered and analyzed the dataset at multiple levels of taxonomic hierarchy (Fig. 2). Nevertheless, we interpret the Viridiplantae rRNA signatures with caution. We also note that as the North Pond sediments are oxygenated all the way to the basement, it is possible that some of the Metazoan rRNA signatures detected at this site derive from living Metazoa.

In order to determine if signals could result from DNA co-extracted with RNA, tests were performed. DNA was not amplifiable from RNA extractions after 40 cycles of PCR, confirming that the majority of DNA co-extracted with RNA was removed during the DNAse treatment, and reduced to mathematically insignificant amounts for amplification in RT-PCR. It is unlikely that remaining trace DNA (if present after the DNAse treatment) in the RNA template for RT-PCR could account for the diatom, metazoan, and plant sequences that represent approximately one-half of the recovered diversity (Fig. 1A, Table 2) in these low eukaryotic biomass environments. We conclude the majority of our dataset is rRNA-derived.

Fossilized diatoms from the genus *Nitzschia* were used to date the Eastern Equatorial Pacific sample (∼2.7mya) [38] and we were able to detect *Nitzschia*-affiliated rRNA from the same horizon, suggesting that this rRNA signal may derive from ancient diatoms that are ∼2.7mya. In this case, the rRNA detected would be 2.7 million years old. While previous studies have reported preservation of DNA in ancient sediments [24], [27] and have suggested the existence of a paleome, our dataset provides the first evidence for ancient eukaryotic rRNA in subseafloor sediments. To identify potentially active subsurface eukaryotes, correlations between broad-ranging taxonomic trends and geochemical factors were investigated.

We divided the dataset into sequences affiliating with the 5 major groups detected: Viridiplantae, Metazoa, Bacillariophyta (Diatoms), Alveolata, and Fungi. Canonical correspondence analyses (CCA) paired with Multi-Response Permutation Procedure (MRPP) at multiple levels of taxonomic hierarchy identified groups correlating with TOC, dissolved inorganic carbon (DIC), sulfide, or sediment depth (Fig. 2 A-E). Sequences from plants showed a high correlation with TOC relative to most other groups, potentially reflecting their detrital origin and contribution to TOC and/or preserved pollen (Fig. 2C). Fungi showed the strongest correlation with TOC across most levels of taxonomic hierarchy (Fig. 2 A, C), consistent with a potentially saprophytic lifestyle. The relatively strong correlation of fungi with DIC (Fig. 2E), plants (Fig. 2C), and TOC (Fig. 2C) may reflect fungal metabolism of organic substrates, and or dead plant material. Subsurface sediments with higher TOC (1.5–3.7 % wt) have 2–3 times more fungal-affiliated sequences compared to samples with lower TOC (0.2–0.01 % wt) (Fig. 1A, B). This is consistent with the statistically significant influence of TOC on determining the composition of subsurface eukaryotic communities across most levels of taxonomic hierarchy [MRPP p value  =  0.02 (+/− 0.01)] (Fig. 2 A). Interestingly, within the Benguela sediment sample two mycorrhizal fungal genera (*Glomus* and *Diversispora*) were detected along with the plant genera (*Plantago* and *Festuca*) that they are typically associated with in terrestrial habitats (Table 2) [39]–[42].

Although TOC, DIC, and sulfide values for the Benguela and North Pond samples were measured from the same sediment depth and general location as the samples from which our rRNA data derive, the measurements were made on separate core samples (values used in the correlation analyses were taken from [43], [44], [45]). Thus to assess the effects of variability on our correlations, we applied TOC, DIC, and sulfide values 2 and 0.5 times the published measurements in separate CCA analyses. This allowed us to assess whether a realistic range of geochemical factors resulted in differential impacts on distributions. Analyses incorporating this range of geochemical values were consistent with the results obtained using the published measurements (Fig. 2), in that in almost every case, Fungi exhibited a correlation (R^2^ value) with TOC, DIC, and sulfide at least twice that of all other groups, suggesting that using proxy geochemical data is sufficient in these locations.

**Figure 3:**
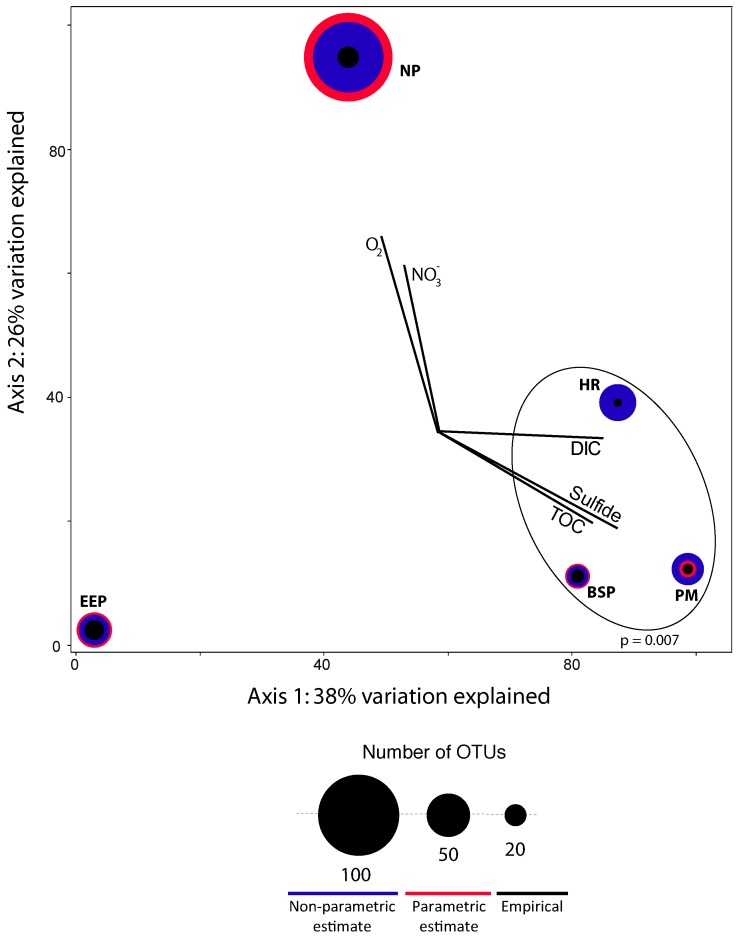
Canonical correspondence analysis of fungal OTUs from marine subsurface samples. Black circles are proportional to the number of fungal OTUs detected in each sample. Red and blue circles are proportional parametric (CatchAll) and nonparametric (Chao1) richness estimates (see Table S2). Vectors represent sulfide, TOC, and DIC correlations. The p-value resulting from an MRPP test for effect of DIC, TOC, and sulfide on fungal distributions is shown in the lower right corner of the biplot. See Table 1 for sample information.

The strong correlation of Fungi with DIC, TOC, and sulfide relative to the other four groups of eukaryotes strongly suggests fungal activity in the subseafloor. These data suggest environmental selection of Fungi in a variety of subsurface oceanic provinces. Further evidence for environmental selection of subsurface fungi comes from the disparity in empirical and estimated fungal richness in anoxic and oxygenated sediments. Fungal richness in the only oxygenated sample in our analyses, North Pond, is estimated to be at least double that of the subseafloor anoxic samples (Fig. 3, Table S3). The correlation of Fungi with nitrate in North Pond sediments (Fig. 3) may indicate fungal utilization of nitrate as a substrate in the subseafloor. Based on this data we suggest the hypothesis that fungal richness in oxygenated subseafloor sediments may exceed that found in anoxic sediments. The fungal communities in anoxic sediments with detectable levels of sulfide are distinct from the community in an anoxic sediment sample with no detectable sulfide (p  =  0.007, Fig. 3), suggesting that the presence of sulfide in anoxic sediments selects for distinct fungal assemblages. In anoxic subsurface samples we found many taxa such as *Candida, Rhodotorula, Rhodosporidium*, and *Trichosporon* (Table 2), known to contain fermentative representatives, albeit *Rhodotorula* contains aerobic species as well. Collectively, this evidence suggests environmental selection of Fungi across a broad range of marine subsurface provinces.

**Figure 4:**
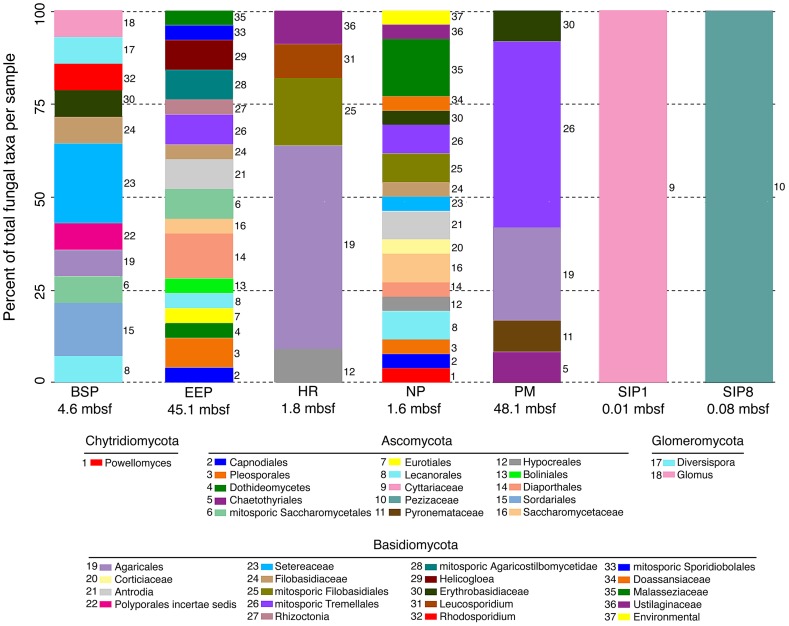
Taxonomic affiliation of fungal OTUs sharing 97% sequence identity from subseafloor and shallow sediment samples. Bar sizes are proportional to taxonomic richness. See Table 1 for sample information.

Comparative samples were included in the analysis, taken from Sippewissett salt marsh, MA, USA, to represent near-surface coastal marine sediments where diversity was hypothesized to be higher than in deeply buried samples. However, the 454 data indicated that the diversity of Fungi might be higher in the deeply buried sediments than in the near surface Sippewissett samples (Fig. 4, Table S3). Because sampling of Sippewissett diversity was limited (Fig. S2, Table S4), we compared subseafloor and near-surface sediment fungal rRNA diversity between samples by applying fungal-specific PCR primers for a terminal restriction fragment length polymorphism (TRFLP) analysis. The fungal-specific TRFLP approach revealed that the Sippewissett near-surface samples actually contain the highest fungal richness of all samples (Fig. S3). Thus, this diversity was missed by the 454 approach, which clearly did not reach saturation for the Sippewissett samples (Fig. S2). However, fungal richness indicated by T-RFLP in three subseafloor samples (Hydrate Ridge, Peru Margin, and North Pond) is comparable to that found in Sippewissett (Figs. S3A, D). Minimal overlap in fungal-affiliated OTUs and TRFLP patterns exists between different deeply buried (>1.5 mbsf) samples, and zero overlap is found between near-surface (<1 mbsf) and deeply buried sediments (Figs. S4 B-D, Fig. S3B). OTUs affiliated with *Mycena*, *Trichosporon, Rhodotorula* and *Cryptococcus* were detected in the majority of subseafloor samples but were absent in the near surface sediments (C). Five similar examples exist in the fungal TRFLP dataset (Fig S4D). Additionally, sediment depth explains a significant amount of fungal TRFLP and rRNA sequence variation (p  =  0.04 and 0.02, respectively) (Fig. 2B). This suggests that many fungal populations in different subseafloor provinces are unique and are distinct from those found in shallow water near-surface marine sediments.

The lack of fungal OTU overlap between shallow and deep samples strongly suggests minimal contamination in this dataset, since no ubiquitous fungal OTUs were found between samples processed in the same laboratory. With confidence that fungal taxa represent sample-specific taxa, we examined potential origins and methods of delivery of subsurface Fungi.

Barriers and rates of microbial dispersal to and from the marine subsurface are not well constrained. Fungi are common as aerosolized spores in dust originating from the deserts of Africa, Asia, and the Middle East, and this dust has been suggested as a possible source of fungal spores to marine sediments [46]. However, only 10% of fungal genera commonly detected in atmospheric samples originating from desert dust [46] were detected in our survey (*Cryptococcus*, *Rhodotorula*, *Neurospora*, *Phoma*, and *Alternaria*). Consistent with the statistically significant influence of sediment depth on most subsurface eukaryotic distributions (Fig. 2B), this suggests the majority of fungi dispersed from deserts into ocean waters via atmospheric movements are not able to penetrate, and/or survive in marine subsurface sediments. While the source of fungi in deep sediments is unknown, physical isolation and environmental selection in the subsurface likely contribute to their phylogenetic uniqueness in different locations.

The existence of dehydrated cellular forms, such as spores, cysts and pollen are known to be widespread in the subsurface and could contribute to the preservation of ancient rRNA. The reduced water content within pollen [47], spores [31], and cysts [48] likely aids the preservation of rRNA within these structures, because the activity of endogenous RNAses decreases under dehydrated conditions [49]. Indeed, many of the taxa detected affiliating with the Diatoms, Viridiplantae, Alveolata, and Fungi (Table 2) are known to produce such structures, and diatom fragments were visualized by calcofluor staining (data not shown). Many of the alveolate-affiliated rRNA reads may derive from dinoflagellate and ciliate cyst-producing taxa detected in our survey. This possibility is further suggested by the detection of aerobic ciliates capable of cyst formation (*e.g. Colpodella* and *Pseudovorticella*) in anoxic sediments dated to be greater than 2 millions of years old (Table 2). This rRNA has likely survived in cyst forms of these ciliates in anoxic sediments for millions of years. As diatoms also produce cysts, this may be the source of some of the diatom-affiliated rRNA detected in our survey, while the rRNA reads affiliated with green plant genera (Table 2) likely derive from buried pollen. The detection of metazoan taxa across the anoxic subsurface samples is surprising because Metazoa are not known, with the exception of rotifers [50], to produce cysts. Thus, the detected metazoan rRNA in anoxic samples derives either from buried detrital metazoan cellular material or from extracellular rRNA. However, the detection of metazoan rRNA in the North Pond sample could be representative of living metazoan cells, as these sediments are oxygenated all the way to basement.

The preservation of extracellular rRNA is a less likely explanation because of the possibility for degradation by extracellular RNAses. However, until preservation of extracellular rRNA in anoxic marine sediments is specifically investigated using methods similar to Corinaldesi et al. (2011), this possibility cannot be ruled out. Consistent with previous reports of preserved extracellular DNA in anoxic marine sediments (e.g. [23]), our results indicate that rRNA extracted from whole sediments cannot be used alone as a universal proxy for living organisms. Thus, rRNA-sequence based molecular approaches should be integrated with geochemical correlations and microscopy, as well as message RNA (metatranscriptomics), to obtain a more realistic assessment of the active fraction of subseafloor microbial communities.

This study shows that ancient rRNA is recoverable and may represent a new tool for paleobiological investigations into the effect of past climate and planetary processes on biological distributions. The study of ancient nucleic acids can be difficult due to the requirement that they be stable for long periods of time, and because interpretation of those sequences in a geochemical context can be complex [51]. Most often ancient DNA is studied in environments where water activity is low or temperatures are freezing. The recovery of 0.3–2.7mya rRNA in marine sediments, an environment that is not arid, crystalline or frozen, suggests this biomolecule may be much more stable than previously considered.

## Supporting Information

Figure S1 **Pie chart showing the representation of the five most abundant eukaryotic taxonomic groups detected.**
(TIF)

Figure S2 **Rarefaction analysis of the 454-pyrosequencing data clustered at 97% sequence identity.** See Table 1 for sample information.
(TIF)

Figure S3 **Abundance of fungal T-RFs within the different samples (A) and the overlap in fungal T-RF**'**s between subsurface and shallow sediments (B).** See Table 1 for sample information.
(TIF)

Figure S4 **Multivariate ordination and heatmap distributions of fungal pyrosequencing and TRFLP data.** (A) Canonical correspondence analysis (CCA) of fungal V4 rRNA OTUs sharing 97% sequence identity. Green and red points represent near-surface and subsurface samples, respectively. Heatmap of fungal OTUs clustered at 90% sequence identity detected across subsurface and shallow sediment samples (B). Sequence abundance within each OTU is log transformed (darker boxes represent more abundant OTUs). (C) CCA of aligned fungal T-RF peaks. (D) Heatmap of aligned fungal T-RF peaks (rows) within sediment samples (columns). Near-surface and subsurface T-RFs are gray and black respectively. See Table 1 for sample information.
(TIF)

Table S1 **The number of reads per sample and the number remaining after quality control and removal of contaminant sequences.**
(DOCX)

Table S2 **Eukaryotic genera affiliated with rRNA sequences deriving from aerosol contaminants.**
(DOCX)

Table S3 **Parametric and non-parametric estimates of fungal richness in subsurface sediments.** See Table 1 for sample information.
(DOCX)

Table S4 **Parametric and non-parametric estimates of eukaryotic richness in subsurface sediments and Sippewissett sediments. See** **Table 1** **for sample information.**
(DOCX)
