# Supplementary figures and images for: Deep Sequencing of Subseafloor Eukaryotic rRNA Reveals Active Fungi across Marine Subsurface Provinces
Source: PLoS One. 2013 Feb 13;8(2):e56335. doi: 10.1371/journal.pone.0056335 (PMC3572030; doi:10.1371/journal.pone.0056335)

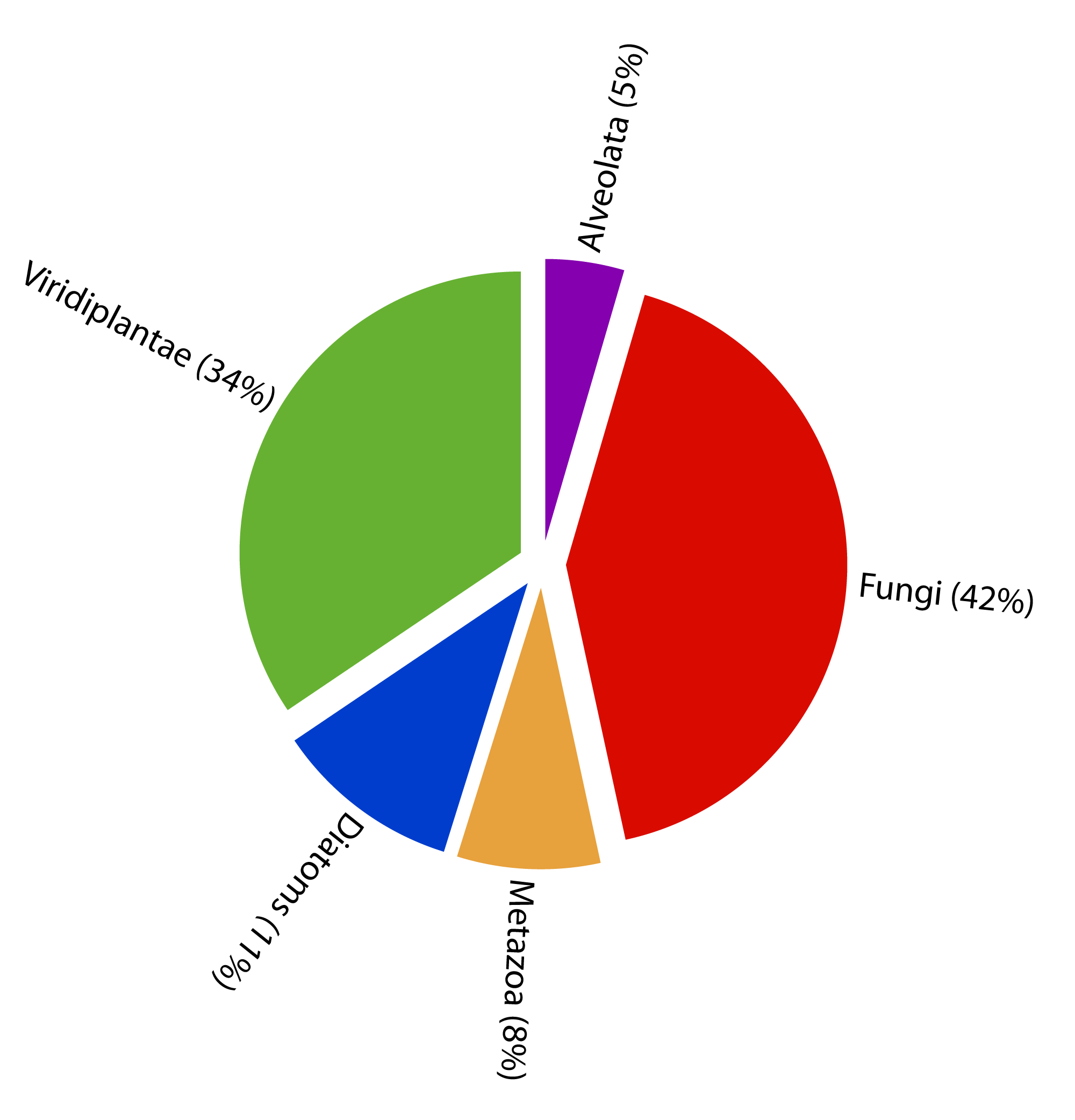

Supplement: Figure S1 — Pie chart showing the representation of the five most abundant eukaryotic taxonomic groups detected. (TIF) [file pone.0056335.s001.tif]

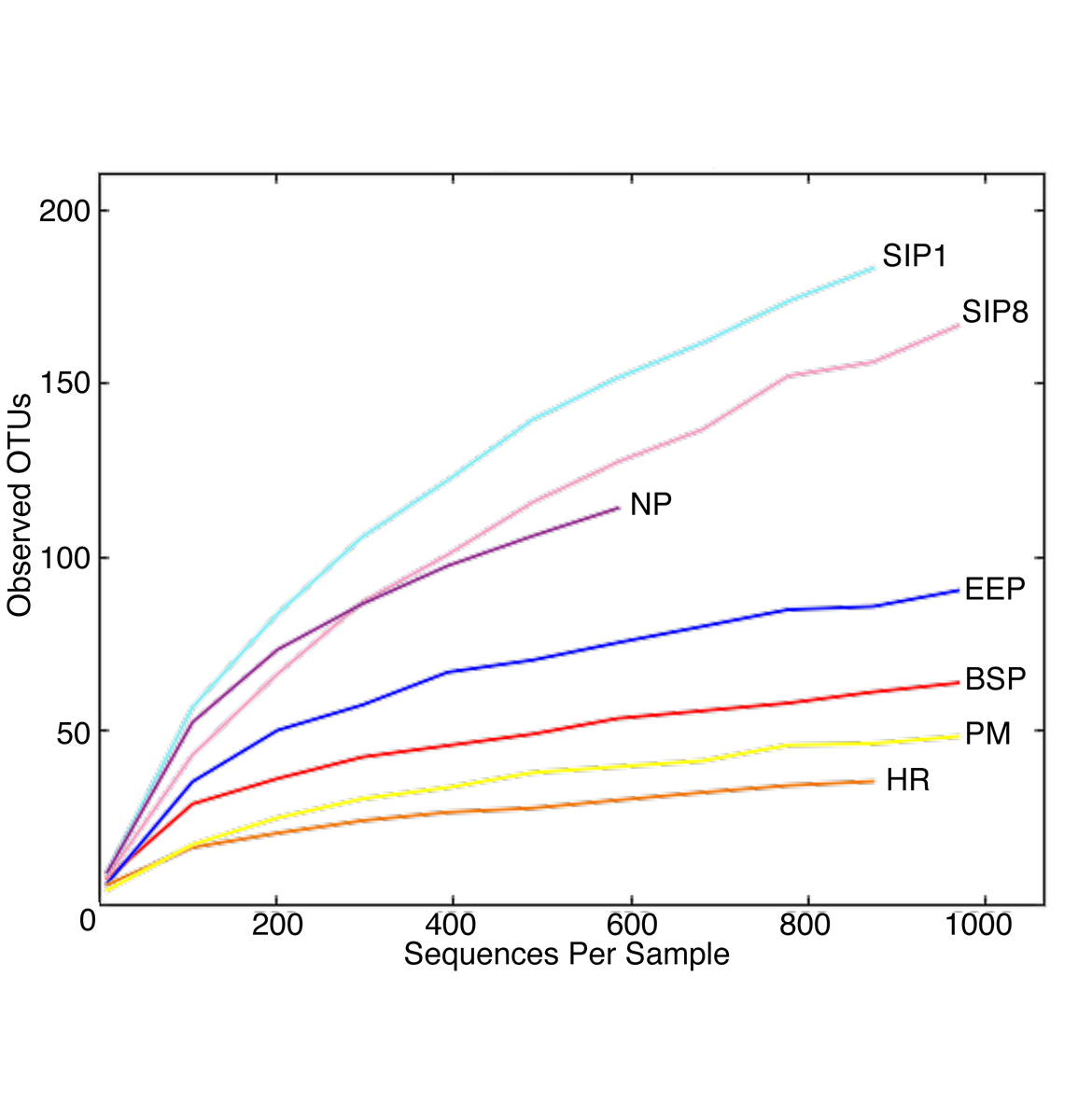

Supplement: Figure S2 — Rarefaction analysis of the 454-pyrosequencing data clustered at 97% sequence identity. See Table 1 for sample information. (TIF) [file pone.0056335.s002.tif]

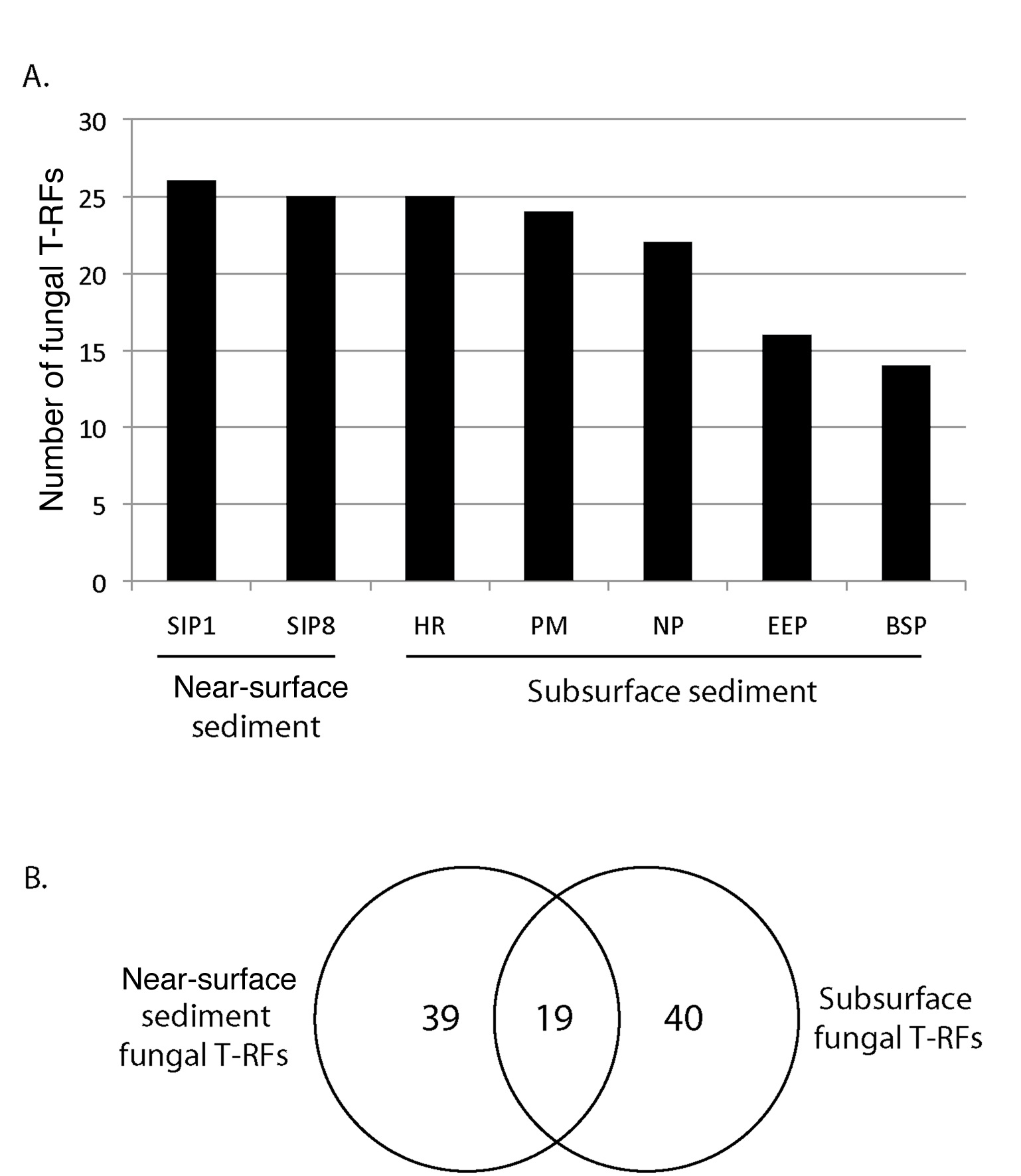

Supplement: Figure S3 — Abundance of fungal T-RFs within the different samples (A) and the overlap in fungal T-RF's between subsurface and shallow sediments (B). See Table 1 for sample information. (TIF) [file pone.0056335.s003.tif]

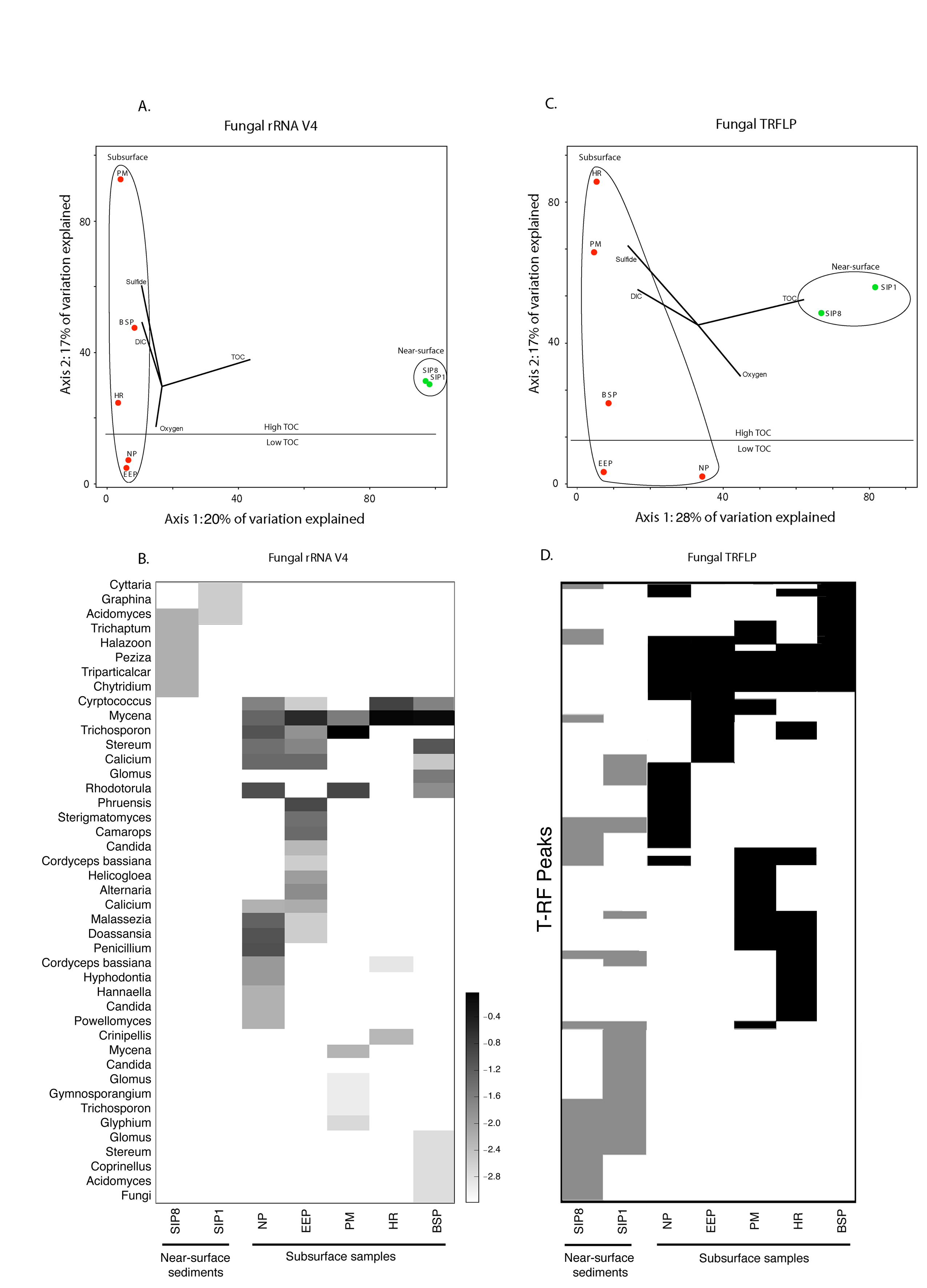

Supplement: Figure S4 — Multivariate ordination and heatmap distributions of fungal pyrosequencing and TRFLP data. (A) Canonical correspondence analysis (CCA) of fungal V4 rRNA OTUs sharing 97% sequence identity. Green and red points represent near-surface and subsurface samples, respectively. Heatmap of fungal OTUs clustered at 90% sequence identity detected across subsurface and shallow sediment samples (B). Sequence abundance within each OTU is log transformed (darker boxes represent more abundant OTUs). (C) CCA of aligned fungal T-RF peaks. (D) Heatmap of aligned fungal T-RF peaks (rows) within sediment samples (columns). Near-surface and subsurface T-RFs are gray and black respectively. See Table 1 for sample information. (TIF) [file pone.0056335.s004.tif]
