# Supplementary material for: Deep Sequencing of Subseafloor Eukaryotic rRNA Reveals Active Fungi across Marine Subsurface Provinces
Source: PLoS One. 2013 Feb 13;8(2):e56335. doi: 10.1371/journal.pone.0056335 (PMC3572030; doi:10.1371/journal.pone.0056335)
Supplement: Table S1 — The number of reads per sample and the number remaining after quality control and removal of contaminant sequences. (DOCX) [file pone.0056335.s006.docx]

| Sample | Depth | Raw reads | Reads passing QC | Reads remaining after removing contaminants |
| --- | --- | --- | --- | --- |
| Sippewissett | 0.01 mbsf | 1441 | 956 | 956 |
| Sippewissett | 0.08 mbsf | 4934 | 3340 | 3336 |
| North Pond | 1.4 mbsf | 3525 | 2180 | 642 |
| Benguela | 4.6 mbsf | 1988 | 1256 | 1092 |
| Hydrate Ridge | 1.8 mbsf | 2737 | 1933 | 950 |
| Eastern Equatorial Pacific | 45.3 mbsf | 11191 | 7567 | 2708 |
| Peru Margin | 48.1 mbsf | 2058 | 1357 | 1001 |
| Total | - | 35429 | 23936 | 10775 |

Table S1
