# Supplementary material for: Deep Sequencing of Subseafloor Eukaryotic rRNA Reveals Active Fungi across Marine Subsurface Provinces
Source: PLoS One. 2013 Feb 13;8(2):e56335. doi: 10.1371/journal.pone.0056335 (PMC3572030; doi:10.1371/journal.pone.0056335)
Supplement: Table S2 — Eukaryotic genera affiliated with rRNA sequences deriving from aerosol contaminants. (DOCX) [file pone.0056335.s007.docx]

| **Fungi** | **Metazoa** | **Viridiplantae** |
| --- | --- | --- |
| *Malassezia Penicillium Saccharomyce Lecanicillium Calicium Cladosporium Pseudozyma Trichocomacea Exophiala* | *Nothodelphax Mythimna Tropidocephala* | *Fagus Pinus Citrus Quercus* |

Table S2
