# Supplementary material for: Deep Sequencing of Subseafloor Eukaryotic rRNA Reveals Active Fungi across Marine Subsurface Provinces
Source: PLoS One. 2013 Feb 13;8(2):e56335. doi: 10.1371/journal.pone.0056335 (PMC3572030; doi:10.1371/journal.pone.0056335)
Supplement: Table S3 — Parametric and non-parametric estimates of fungal richness in subsurface sediments. See Table 1 for sample information. (DOCX) [file pone.0056335.s008.docx]

| Sample | EEP 45mbsf | PM 48mbsf | BSP 4.6mbsf | HR 1.8mbsf | NP 1.6mbsf |
| --- | --- | --- | --- | --- | --- |
| Best Parametric Model | Single exponential | Single exponential | Single exponential | NA | Single exponential |
| Observed OTUs (97% identical) | 24 | 12 | 16 | 9 | 26 |
| Estimated total number of OTUs (SE) | 32 (+/- 4) | 14 (+/- 2) | 20 (+/- 2) | NA | 63 (+/-17) |
| 95% Confidence Interval | 27-44 | 13-20 | 17-28 | NA | 42-112 |
| Goodness-of-fit | 0.18 | 0.5 | 0.42 | NA | 0.5 |
| Chao1 | 64 (+/- 49) | 22 (+/- 9) | 26 (+/- 9) | NA | 35 (+/- 7) |
| ACE1 | 32 (+/- 6) | 24 (+/- 16) | 21 (+/- 5) | 24 (+/- 22) | 63 (+/- 26) |

Table S3
