# Supplementary material for: Deep Sequencing of Subseafloor Eukaryotic rRNA Reveals Active Fungi across Marine Subsurface Provinces
Source: PLoS One. 2013 Feb 13;8(2):e56335. doi: 10.1371/journal.pone.0056335 (PMC3572030; doi:10.1371/journal.pone.0056335)
Supplement: Table S4 — Parametric and non-parametric estimates of eukaryotic richness in subsurface sediments and Sippewissett sediments. See Table 1 for sample information. (DOCX) [file pone.0056335.s009.docx]

| Sample | EEP 45mbsf | PM 48mbsf | BSP 4.6mbsf | HR 1.8mbsf | NP 1.6mbsf | SIP1 0.01mbsf | SIP8 0.08mbsf |
| --- | --- | --- | --- | --- | --- | --- | --- |
| Best Parametric Model | Single exponential | Two-mixed exponential | Single exponential | Single exponential | Single exponential | Two-mixed exponential | Two-mixed exponential |
| Observed OTUs  (97% identical) | 74 | 35 | 51 | 33 | 118 | 159 | 128 |
| Estimated total number of OTUs (SE) | 95 (+/- 6) | 59 (+/- 10) | 62 (+/- 4) | 47 (+/- 8) | 186(+/-14) | 364 (+/- 49) | 274 (+/-34) |
| 95% Confidence Interval | 86-110 | 46-86 | 56-74 | 38-73 | 163-220 | 288-486 | 221-358 |
| Goodness-of-fit | 0.02 | 0.23 | 0.03 | 0.34 | 0.34 | 0.07 | 0.6 |
| Chao1 | 116(+/-23) | 91(+/- 49) | 77(+/- 18) | 43(+/- 8) | 179(+/-25) | 332(+/- 56) | 222(+/- 32) |
| ACE1 | 100(+/-11) | 67(+/- 57) | 70(+/- 10) | 80(+/- 34) | 165(+/-15) | 394(+/- 76) | 220(+/- 26) |
| % of predicted taxa detected | 78(+/- 8) | 59(+/-17) | 82(+/- 9) | 70(+/- 16) | 63(+/- 12) | 44(+/- 11) | 47(+/- 11) |

Table S4
